# Supplementary material for: Ascorbate Plus Buformin in AML: A Metabolic Targeted Treatment
Source: Cancers (Basel). 2022 May 23;14(10):2565. doi: 10.3390/cancers14102565 (PMC9139619; doi:10.3390/cancers14102565)
Supplement: Supplementary file 1 [file cancers-14-02565-s001.zip › cancers-1722573-supplementary.pdf]

Supplementary Figure S1

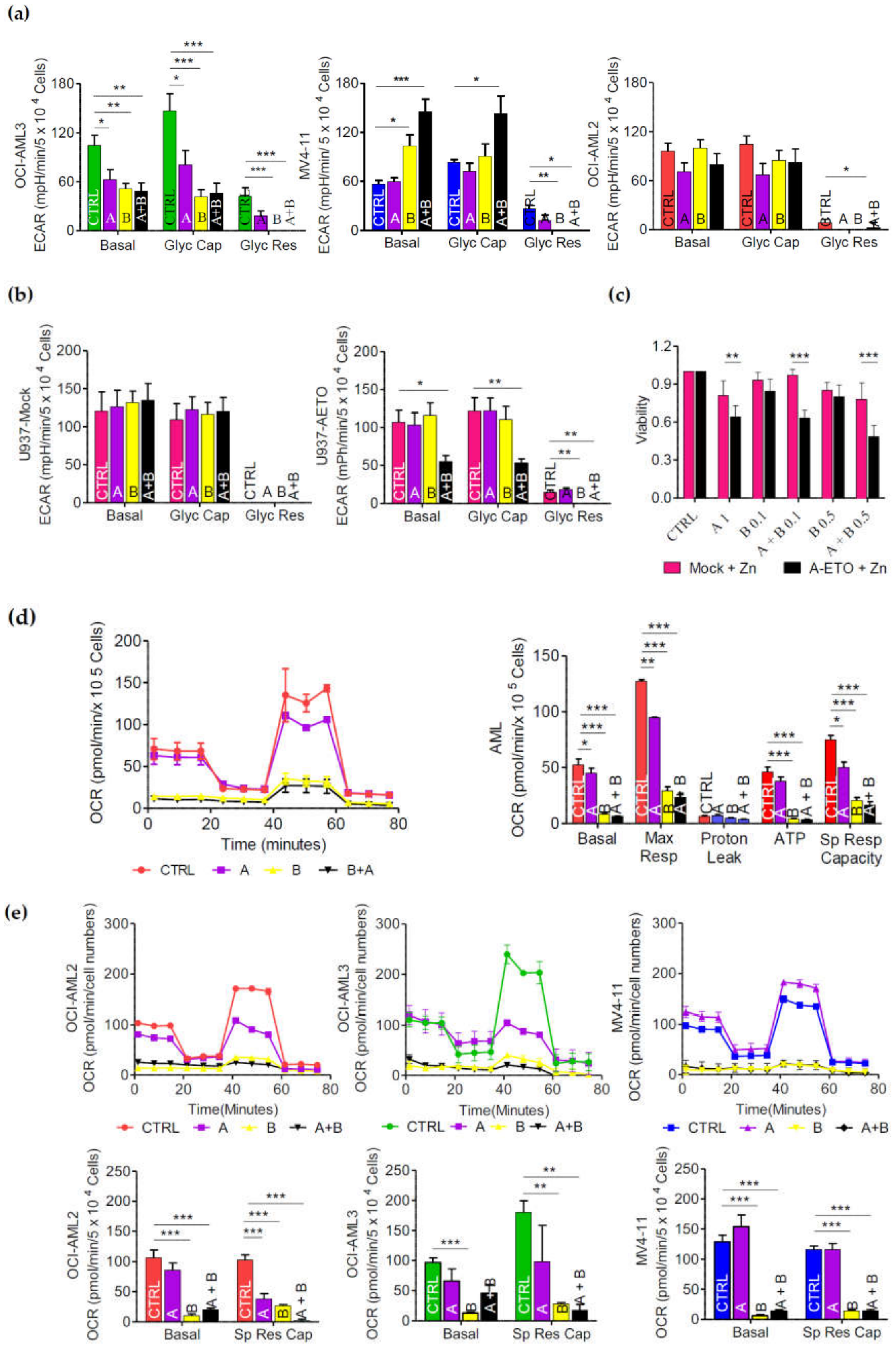

(f)

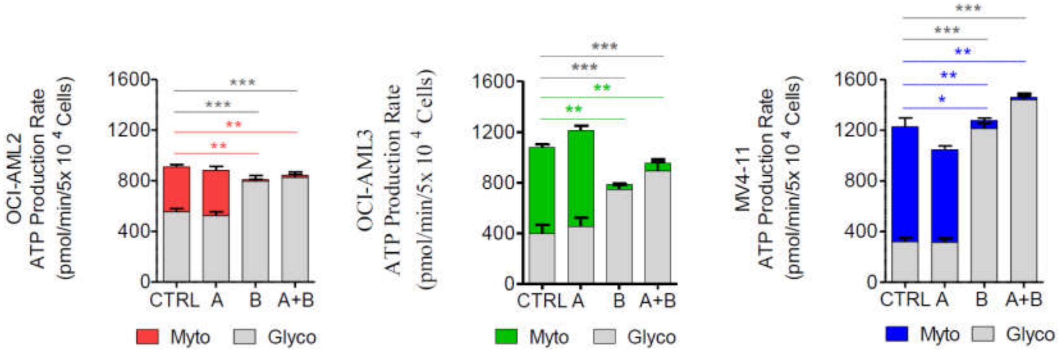

Supplementary Figure S1. Metabolic effect of buformin and ascorbate in AML cells. Cell lines were treated for 24 hours with 1mM ascorbate (A), 0.1 mM buformin (B) or the ascorbate-buformin combination and evaluated by XF Glycolytic Stress Test. Histograms represent Basal Glycolysis, Glycolytic Capacity (Glyc Cap) and Glycolytic Reserve (Glyc Res) (**a**) OCI-AML3, MV4-11 and OCI-AML2, (**b**) U937-Mock and U937-AETO. (**c**) Cytotoxic efficacy of 1Mm ascorbate (A), 0.1 and 0.5 mM buformin (B) or the ascorbate-buformin combinations in U937-Mock and U93-AETO using the MTS assay test. Data are presented as mean ± SD. Statistical analysis by the Anova t test and Tukey's multiple comparison test. *p<0,05; **p<0,005; ***p<0.0005. (**d**) AML cells treated for 24 hours with 1mM ascorbate (A), 0.1 mM buformin (B) or the ascorbate-buformin combination and evaluated by XF Myto Stress Test. (**e**) Histograms represent basal respiration, maximal respiration, proton leak, ATP and spare respiratory capacity (Spare Res Cap). (e) OCI-AML2, OCI-AML3 and MV4-11 cell lines treated for 24 hours with 1mM ascorbate (A), 0.1 mM buformin (B) or the ascorbate-buformin combination and evaluated by XF Myto Stress Test. (e) Histograms represent basal respiration and spare respiratory capacity (Spare Res Cap). (**f**) XF real-time glycolytic and mitochondrial ATP production rate by the ATP rate assay. ATP production after 12 hours of 1mM ascorbate (A), 0.1 mM buformin (B) or the ascorbatebuformin combination in OCI-AML2, OCI-AML3 and MV4-11. Statistical analysis by Student's t-test.

Supplementary Figure S2

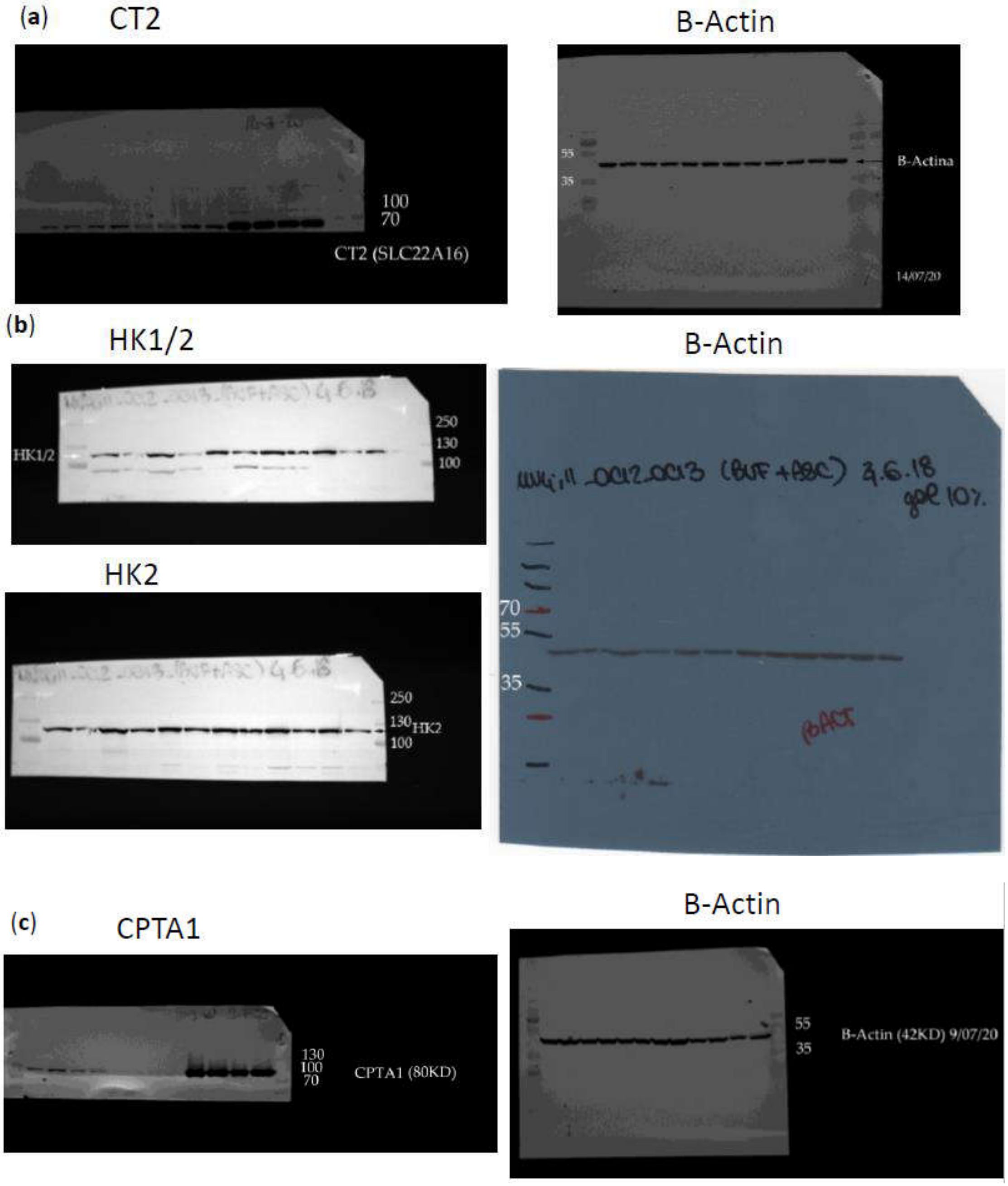

(**d**)

Glut 1 B-Actin

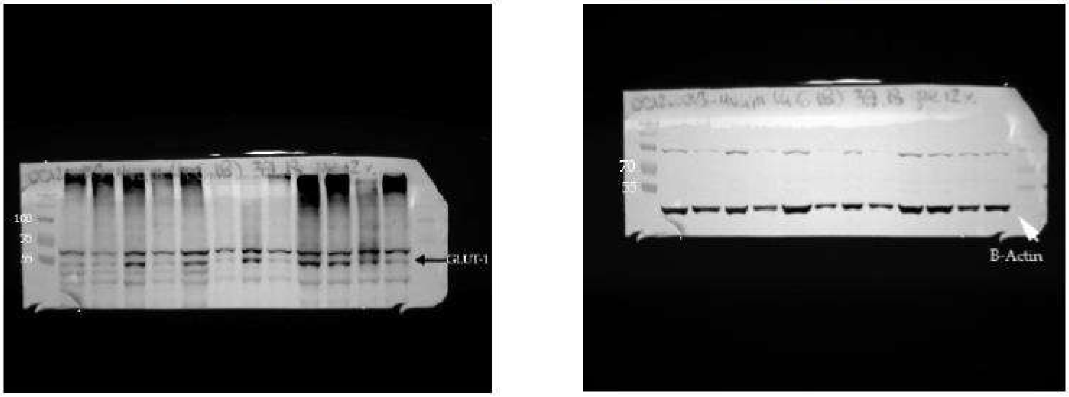

Supplementary Figure S2. Original western blots of protein expression in OCI-AML2, OCI-AML3 and MV4-11 cell lines treated with 1 mM of ascorbate, 0.1 mM of buformin or ascorbate–buformin combination (**a**) CT2 (left panel) and B-Actin (right panel); (**b**) Hexokinase 1 and hexokinase 2 (HK1/2) (upper left panel), hexokinase 2 (low left panel) and B-Actin (right panel); (**c**) CPTA1 (left panel) and B-Actin (right panel); (**d**) Glut1 (left panel) and B-Actin (right panel).

Table S1. List of primary antibodies used.

HK2 (rabbit)	22029-1-AP	Proteintech, Naperville, IL, USA
HK1/2 (mouse)	MAB8179	R&D System, Minneapolis, MN, USA
Glut-1 (rabbit)	07-1401	Millipore, Temecula, CA, USA
CPT1-A (mouse)	8F6AE9	Abcam, Boston, MA, USA
CT2 (SLC22A16) (rabbit)	NBP1-85410	Nobus biologica, cambridge, UK
B-Actin (mouse)	3700	Cell Signaling, Danvers, MA (USA)
